# Integration of Gravitational Torques in Cerebellar Pathways Allows for the Dynamic Inverse Computation of Vertical Pointing Movements of a Robot Arm

**DOI:** 10.1371/journal.pone.0005176

**Published:** 2009-04-22

**Authors:** Rodolphe J. Gentili, Charalambos Papaxanthis, Mehdi Ebadzadeh, Selim Eskiizmirliler, Sofiane Ouanezar, Christian Darlot

**Affiliations:** 1 CNRS UMR 7060, Université Paris Descartes, Paris-5, Paris, France; 2 Université Paris Diderot, Paris-7, Paris, France; 3 INSERM U887, Motricité-Plasticité, Université de Bourgogne, Dijon, France; 4 Ecole supérieure des Télécommunications, Paris, France; 5 Amirkabir University of Technology, Computer Engineering and Information Technology Department, Tehran, Iran; 6 Cognitive Motor Neuroscience laboratory, Department of Kinesiology, University of Maryland, School of Public Health, College Park, Maryland, United States of America; L'université Pierre et Marie Curie, France

## Abstract

**Background:**

Several authors suggested that gravitational forces are centrally represented in the brain for planning, control and sensorimotor predictions of movements. Furthermore, some studies proposed that the cerebellum computes the inverse dynamics (internal inverse model) whereas others suggested that it computes sensorimotor predictions (internal forward model).

**Methodology/Principal Findings:**

This study proposes a model of cerebellar pathways deduced from both biological and physical constraints. The model learns the dynamic inverse computation of the effect of gravitational torques from its sensorimotor predictions without calculating an explicit inverse computation. By using supervised learning, this model learns to control an anthropomorphic robot arm actuated by two antagonists McKibben artificial muscles. This was achieved by using internal parallel feedback loops containing neural networks which anticipate the sensorimotor consequences of the neural commands. The artificial neural networks architecture was similar to the large-scale connectivity of the cerebellar cortex. Movements in the sagittal plane were performed during three sessions combining different initial positions, amplitudes and directions of movements to vary the effects of the gravitational torques applied to the robotic arm. The results show that this model acquired an internal representation of the gravitational effects during vertical arm pointing movements.

**Conclusions/Significance:**

This is consistent with the proposal that the cerebellar cortex contains an internal representation of gravitational torques which is encoded through a learning process. Furthermore, this model suggests that the cerebellum performs the inverse dynamics computation based on sensorimotor predictions. This highlights the importance of sensorimotor predictions of gravitational torques acting on upper limb movements performed in the gravitational field.

## Introduction

How the mechanical effects of gravity (gravitational torques), exerted on a stationary or a moving limb are processed by the Central Nervous System (CNS) is an important question in motor control. In particular, it has been suggested that such mechanical effects on the sensorimotor system are centrally represented in the brain in internal models [1], [2]. One proposal was that the brain uses internal models incorporating the dynamics of the gravitational field acting on moving objects [2]. Particularly, it has been suggested that the CNS uses an internal model of gravity to predict gravitational acceleration allowing the subjects to intercept falling objects in the gravitational field [3]. Another research line analyzed kinematics and dynamics of vertical arm movements performed under normal or altered gravity conditions to examine how the brain deals with gravity during motor planning and control. Specifically, some authors proposed that the interaction of the gravitational field with the motor system is centrally integrated by the CNS and used during motor planning to take advantage of the gravity force to decelerate upward and accelerate downward arm movements [4], [5]. Similarly, there is evidence of a common coordinated strategy involving a muscular deactivation/activation set during rapid leg flexion suggesting that the brain uses gravitational effects to initiate and brake leg motion [6].

In computational motor control several studies have investigated internal models (inverse, forward) by manipulating mechanical constraints. The internal forward model predicts the future states of the limb by using an efferent copy of the neural command whereas the inverse model inverts the causal flow by computing the neural command from a desired movement. Thus, the inertia would be integrated into internal models of limb biomechanics and therefore accurately predicted during arm movements [7], [8].

Although many investigations focused on these internal models, some questions remain such as which type of internal model might be implemented in a particular neural structure. Some authors proposed that the cerebellum implements an inverse model, performing therefore the dynamic inverse computations [9], [10] whereas others investigations argued that the cerebellum generates sensorimotor predictions through an internal forward model [11], [12]. Interestingly, adaptation studies of arm movements showed that the cerebellum takes into account the dynamics and kinematics of motion [13], [14]. However, no theoretical or experimental investigation examined whether the cerebellum incorporates a neural network able to encode the interactions of the limbs with the gravitational field, namely an internal model of gravitational torques. If such an internal model exists, is it based on inverse computations or, alternatively, on sensorimotor predictions? The aim of this study was to propose a model of cerebellar pathways that performs the dynamic inverse computation of the gravitational effects from its sensorimotor predictions, and to assess whether it allows controlling an artificial anthropomorphic robot arm performing pointing movements in the sagittal plane.

## Materials and Methods

The model of the cerebellar pathway presented here is derived from that previously proposed [15], [16]. These authors have exclusively considered horizontal pointing movements executed by a robotic arm. The present study considers the situation where the robotic limb was subjected to various gravitational effects resulting from specific combinations of movement directions, initial positions and amplitudes during pointing movements in the sagittal plane. Indeed, such combinations impose important quantitative and qualitative differences in the gravitational torques exerted on the arm [5].

### The model

From a computational point of view, the calculation of a motor signal, such that the executed movement equals the desired one, requires the biophysical features of the limb to be integrated into the pre-motor circuits. Not only the biological properties of the muscles (e.g. stiffness, viscosity) must be considered, but also the inertial properties of each limb, the reciprocal interaction forces between segments and their interactions with external forces such as gravity.

**Figure 1:**
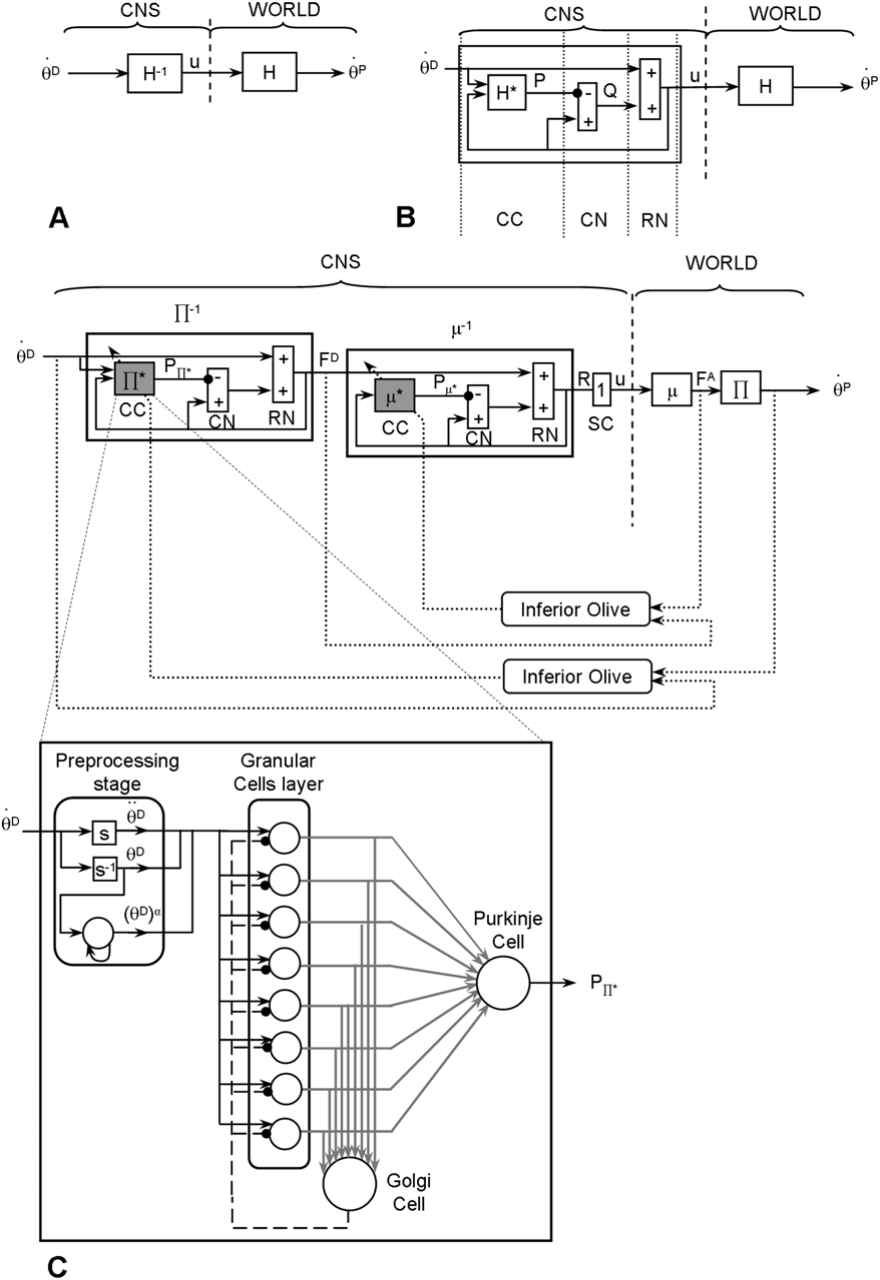
Main principles of the cerebellar architecture of the model. For the sake of clarity, only the command circuit for one muscle is illustrated. (A) Structure of a command circuit accounting for the physical constraints. θ^D^: desired movement; θ^P^: performed movement; u: neural command. H: direct function incorporating all biophysical features of the limb. H^−1^: internal inverse model of the direct function. (B) Control scheme used to compute an approximate inverse function. The two summing elements (positive/negative inputs) represent the cerebellar nuclei (CN) and the red nucleus (RN). H*: internal forward model of the direct function H. P and Q represent the signals originating from the cerebellar cortex (CC) and CN, respectively. (C) Direct functions Π* (representing the mechanical constraints, e.g. gravity, inertia) and μ (representing the muscle features) in the external world, and their counterparts in the CNS labeled Π* and μ*. These two internal forward models (Π* and μ*) are embedded through two internal feedback loops placed in series to calculate their approximate inverse, i.e., Π^−1^and μ^−1^. The direct pathways convey signals of desired position θ^D^ and forces F^D^. In the indirect pathways, the negative output of the elements computing Π* and μ* are comparable to the inhibitory projections of the Purkinje cells of the cerebellar cortex to the neurones of the cerebellar nuclei. The P signals are comparable to simple spike activities of Purkinje cells. Dashed lines represent the climbing fibers. SC: spinal cord. Lower scheme: artificial neural networks simulating the CC. Granular, Golgi and Purkinje cells (respectively 8, 1 and 1 for each predictor) are modelled by formal neurones. s and s^−1^ represent respectively the derivative and integration in the Laplace domain. α represents multiplicative higher orders of the position. The adaptive elements and connections are represented in grey, fixed elements in black. For the sake of the clarity only the first neural network is represented.

This could be achieved by means of internal inverse models of the biophysics of the moving limbs, embedded in neural networks [16]–[18] (Figure 1A). Such an inverse computation can be performed by a neural circuit (Figure 1B) composed of two parallel, closed, internal feedback loops: a positive one (with a gain close to, but smaller, than one to insure stability) and a negative one containing an internal forward model of the direct biophysical function of the limb (denoted H* in Figure 1B), that processes motor commands and computes predictive signals anticipating sensorimotor signals [15], [16], [19].

This circuit, by means of its structure formed of two short and parallel feedback loops, one of which containing a predictor, computes an approximate internal inverse model of the direct biophysical function of the limb. This is nevertheless achieved without performing an explicit inverse calculation. Together, these two loops provide a feed-forward control. From an anatomical viewpoint, the internal forward model H* is thought to correspond to the cerebellar cortex, which receives sensory signals (Figure 1B, C) as well as efferent copies of motor commands (u) through the mossy fibers. The resulting Purkinje cell activity represents the simple-spike inhibitory signal that is sent to the cerebellar nuclei, and is thought to predict torque based on the motor command. This inhibitory signal, together with the efferent copy of the neural command (u), is then fed to a summing element which would represent the cerebellar nuclei (labeled CN in Figure 1B,C). The efferent copy loops through a second (downstream) summing element (labelled RN in Figure 1B–C) which represents the magno-cellular part of the red nucleus. This last summing element adds the output signal issued from the summing element representing the cerebellar nuclei to the signal coding the desired movement. It is noticeable that the motor command (u) reaches both the predictor and this summing element, similar to the messages conveyed by the excitatory mossy fibers that reach both the cerebellar cortex and the cerebellar nuclei.

Both the inverse kinematics and the inverse dynamics problem need to be solved in order to compute an adequate neural command. However, since the robotic arm used in our experiment has only one geometrical degree of freedom (DoF), the inverse kinematic problem is not of interest here. Nevertheless, the robotic arm is actuated by a pair of antagonist muscles which requires the computation of the inverse dynamics at two stages: first, the inversion of the biophysical characteristics of the muscles (including viscosity and stiffness) and second the inversion of the biomechanics of the moving segment, (including the physical constraints applied to the arm such as the gravitational and inertial torques). Notably, these inverse computations must deal with a combination of non-linearities due to the actuators and due to the different movement directions (up, down) in the gravitational field.

The motor command (denoted u_i_) computed in this model (Figure 1A–C) can be compared to the activity of a pool of motoneurons allowing muscular contractions of the i^th^ muscle, which produces a force f_i_, by means of a biophysical process described by the direct function denoted μ_i_. It must be noted that here i ∈{1,2} since we consider two muscles (for the sake of clarity, in Figure 1 we used simplified notation since only one circuit command allowing to compute the motor command for one muscle is illustrated). Then, these forces act on the joint and produce the resulting torque to accelerate the movable segment. Here, the torque T_i_ is related to the force f_i_ with respect to the rotation centre O, according to the following equation:
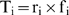
(1)where “×” denotes the cross product (for simplicity arrows above vectors have been omitted) of two vectors r_i_, whose origin is the insertion point of the muscle and whose extremity is the point of application of the force f_i_ on the moving limb. According to Newton's law, the equation expressing the total torque exerted at the joint by the actuators and the mechanical forces applied to the one DoF arm moving in the sagittal plane is as follow:
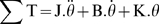
(2)

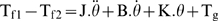
(3)

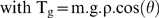
(4)where T_f1_ and T_f2_ are the respective torques developed by the muscle 1 and 2. J is the moment of inertia and 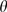, 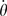 and 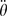, are respectively the angular position, velocity and acceleration of the limb. B and K denote respectively the coefficients of viscosity and stiffness of the joint. Finally, m, g and ρ denote respectively the mass of the limb, the gravitational acceleration and the radius drawn from the axis of rotation to the gravity vector. The direct transfer function of the dynamic mechanical constraints (e.g. gravity, inertia) applied to the limb are denoted by Π (Figure 1C). It must be noted that the direct functions μ and Π act in series, since the first one provides the force exerted by the muscles (given the neural command), whereas the second provides the movement caused by the torque resulting from the various forces applied to the arm (see the right side of Figure 1C, labeled “World”). Elaboration of the motor commands requires the successive computations, in the reverse order, first of the approximate inverse function of Π (labelled Π^−1^), which provides the desired force from the desired movement, and second of the approximate inverse function of μ, (labelled μ^−1^), which provides the neural command from the desired force (see the left part, labeled “CNS”, of Figure 1C).

The inverse computation of these two functions μ^−1^ and Π^−1^ is performed by means of the general computational scheme depicted in Figure 1B. Therefore, the neural control circuit shown in Figure 1C includes two internal forward models (denoted μ* and Π* mimicking respectively the two direct functions μ and Π in order to predict the desired movement and force) put in series and embedded within two distinct cerebellar neural network modules. The predictors μ* and Π* were modelled using artificial neural networks whose architecture was designed by replicating the well-known connectivity of the cerebellar cortex [15], [16] (for reviews see [20], [21]).

#### Connectivity of the neural networks

The three principal types of cells of the cerebellar cortex, i.e. the granular, Golgi and Purkinje cells, were modeled by means of formal neurons (Figure 1C). Convergence of various afferent messages onto neurons was modeled as a weighted algebraic sum of input signals. A first order differential equation, described in the Laplace domain by a low pass filter with a time constant of 5 ms, representing the recruitment was used for the input function of the neurons. Granular, Golgi and Purkinje cells were assumed to act as low-pass filters, with time constants of 10, 5 and 5 ms, respectively. Their activation functions were modeled by a sigmoid accounting for saturation of neuronal activity. The proportions of the various cell types were not respected, since there were very few granular cells compared to Golgi and Purkinje cells (for each predictors n = 8, n = 1 and n = 1, respectively). The input to the two predictors Π* and μ* (based on an identical internal architecture) were respectively the desired angular velocity and the desired force. For instance, for an arm movement of 25°, the first predictor (Π*) received the desired angular velocity for this movement and then computed the corresponded desired force for each muscles which represented the output signal of this first predictor. Then these desired forces were used as inputs for the second predictor (μ*) that computed the corresponding neural command. These inputs signals were transmitted by the granular cells and their mossy fibers. These signals, including a feedback via the Golgi cells, were conveyed to the Purkinje cells via the parallel fibers. The parallel fiber - Purkinje cell connections, whose weights were adjusted by means of a learning process, represented the main learning sites of this neural network. (see Figure 1C, adaptive elements are shown in gray). A minor difference between these two predictors was that, upstream, the granular cells, the architecture of the predictor Π* included an additional processing stage (labeled preprocessing layer) such simple operations could be done for instance in pre-cerebellar nuclei or in the glomeruli (Figure 1C). Computations applied to the desired angular velocity signal allowed computing higher multiplicative orders (e.g. squared functions) and integral or derivative terms. Granular cells were thus provided with a variety of signals which contributed to the signals that they processed and that were encoded in parallel fibers. Such a variety of dynamic signals allowed representing accurately, within the neural network, the non-linearities of the mechanics of the moving segment and of the muscles. Compared to the previous model [16], the number of the granular cells has been increased, to permit to take into account the mechanical effects of the gravity force. The efficiency of this model has been first tested by simulations and then by robot experiments.

#### Motor learning

The synapses between parallel fibers and Purkinje cells are well-known neural sites of plasticity in the cerebellum, modulated by teaching signals arising from the Inferior Olive and conveyed via climbing fibers. A supervised learning procedure schematizing this circuitry was incorporated into our model. Based on previously results, no other sites of cerebellar plasticity were introduced [15], [18]. Learning was modeled by modifying progressively the input weights of the parallel fiber signals, according to the correlation of these signals with a teaching signal calculated from the quadratic error between the desired and performed positions and forces (Figure 1C).
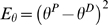
(5)

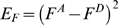
(6)


**Figure 2:**
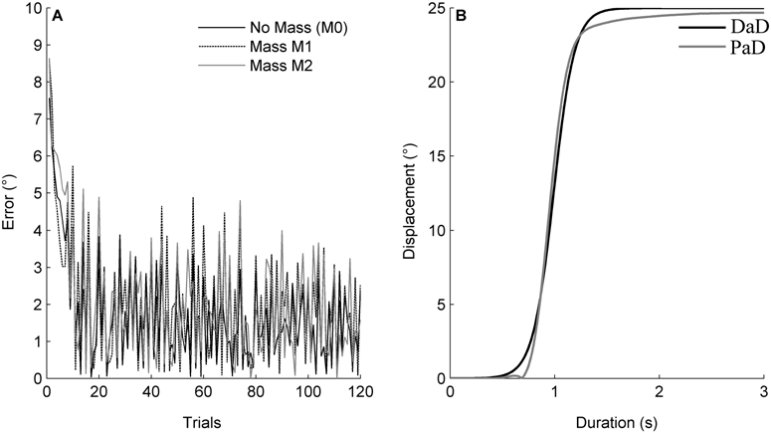
Learning curves for robotic experiment. (A) Three learning curves obtained for the three mass conditions M0, M1 and M2. (B) Typical desired and performed arm displacements from a horizontal position (i.e. 0°) and with an amplitude of 25°. DaD: Desired angular Displacement. PaD: Performed angular Displacement.

A teaching signal was sent whenever the difference in position was superior to 0.5° (simulated) or to 1° (for robot movements, Figure 2B) and the difference in force >20 g according to the following learning rules:
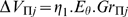
(7)

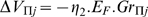
(8)
*where* 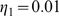 and 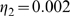.

Details and demonstrations of the learning rules are given in [16], [18]. Typical learning curves with an asymptotic profile are shown in Figure 2A.

### Experimental design

#### The robotic limb

The robot used for the experiments consisted of a single movable segment actuated by two antagonist pneumatic muscles (for details see [15], [16]). Briefly, two similar artificial McKibben muscles pulled oppositely on the ends of a chain engaged in a sprocket rotating around the horizontal axis and integral to one tip of the segment. The lever arm was constant and equal to the radius of the sprocket. Thus, the angular movement resulted from the difference between the forces exerted by the two muscles. The force exerted by the two muscles, depended in turn on the pressure in the muscle and also on the current muscle length and elasticity. Pressures in the muscles were independently set by two servo-valves driven by a computer using a digital analog converter. Therefore, the overall system can be considered as an approximation of the human upper limb (the shoulder joint with two muscles: deltoid anterior and posterior), since it is composed of a pair of antagonist muscles, although artificial, with a rotating sprocket. This movable segment had one geometrical DoF and two dynamical DoF, since the two forces exerted by the muscles were independent from each other. The segment rotated by an angle θ when the two muscles were inflated at different pressures. Noting respectively p_1_ and f_1_ the pressure and force of the agonist muscle, and p_2_ and f_2_ for of the antagonist, r being the sprocket radius, l_0_ the muscle length at rest, and ε_1_ and ε_2_ their contraction ratios (depending of the neural drive u_i_), the resultant torque T was produced by the muscles according to the following relationships [22]:
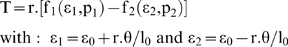
(9)


The angular position of the segment was measured by means of a potentiometer having a precision of 1 degree. Muscle tensions were measured by means of force sensors placed at one end of each muscle. Noise in the potentiometer and in the force sensors was filtered out by means of low-pass filters put in series, having time constants of 0.1 second.

#### Motor task and gravitational constraints applied to the robot arm

**Figure 3:**
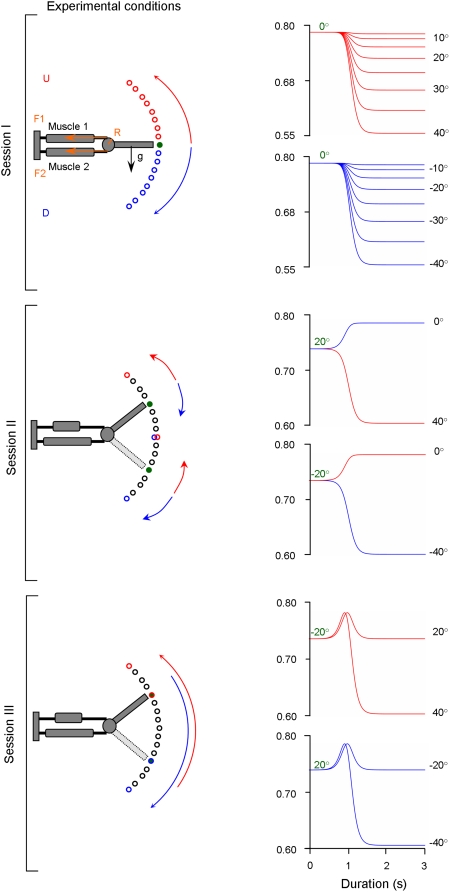
Schematic explanation of the setup and the three simulated tasks. Representation of the simulated arm and its two antagonist muscles. F1: force developed by muscle 1. F2: force developed by muscle 2. R: radius of the sprocket. Black arrow: gravitational torque (GT) exerted on the segment. First row: Session I. Initial position (green circle) at 0°; Required movements: upward (red arrow), downward (blue arrow). Traces to the right: gravitational torque over time as a function of target movement amplitude and direction (upward: red, downward: blue). For all movements of session I, the gravitational torque varies monotonically and is independent of movement direction. Second row: Session II. Initial positions at 20° and −20°. The gravitational torque varies monotonically but depends on movement direction. Third row: Session III. Initial positions at 20° and −20°. For each movement (amplitudes: 40° and 60°) the gravitational torque varies non-monotonically.

Specific combinations of movement direction, initial position and amplitude of limb motion (i.e. joint angle) in the sagittal plane can lead to important non-linear variations on the gravitational effects exerted on the limb [5]. Therefore, the dynamics of the limb cannot be computed with straightforward classical command methods or by a simple inverse calculus. As a consequence, in order to test the learning capacities of this cerebellar model to compute the inverse dynamics of gravity torques, we manipulated the gravitational effects applied to the arm through three experimental sessions during which various initial positions, movement amplitudes and directions were combined during vertical arm pointing movements. For both the simulated and the real robotic limb, vertical arm movements were performed to specific targets (Figure 3): up to 40°, 35°, 30°, 25°, 20°, 15°, 10°, 5°, 0°, and down to −5°, −10°, −15°, −20°, −25°, −30°, −35°, −40°. The origin (0°) of this frame of reference was located at the axis of rotation of the robotic arm, which corresponds to the human shoulder joint level. Figure 3 illustrates the three behavioral sessions; each session is distinguished by a particular variation of the gravitational torques.

In the first session, all movements started from the same initial position, namely at 0°. Eight upward (i.e. 5°, 10°, … 40°) and eight downward (i.e. −5°, −10°, … −40°) pointing movements towards targets at multiples of 5° were performed. Under these conditions, the gravitational torque is a decreasing monotonous function of movement amplitude, but is independent of movement direction (Figure 3A, compare red and blue gravitational torques traces). Thus, the gravitational torque is identical for up and down movements of similar amplitude, although the arm moves against gravity (upward movements) and with gravity (downward movements).

In the second session (Figure 3B), arm movements were performed from the initial positions of 20° and −20°. From these initial positions, the arm had to perform 20° rotations, i.e. from the initial position of 20° down to 0° or up to 40° and from the initial position of −20° up to 0° or down to −40°. Under these conditions, the gravitational torque is a function of movement amplitude and direction, as well as of the initial position, and varies for a given direction in a monotonous fashion. For example, from the initial position of 20°, a downward displacement implies an increase in gravitational torque, whereas the same movement started from the −20° position involves a decrease in gravitational torque. In addition, the amplitude of these variations is not only opposite but also differs in magnitude. For instance, from the 20° initial position, when the arm moves upward and downward, the gravitational torques decrease and increase, respectively (i.e. opposite changes); in addition, the amounts of these changes were different (due to the non linearity of the gravitational torque). Since the initial arm positions of 20° and −20° are symmetrical with respect to the horizontal axis, gravitational torque values are identical at movement onset for movement in session II.

In the third session (Figure 3C), arm pointing movements were performed from the same initial positions as in session II, however, movement amplitudes were at least twice as large (i.e. 40°, 60°). Under these conditions, the gravitational torque varies again as a function of movement amplitude, direction and initial position, but now in a non-monotonous fashion since the arm crosses the horizontal plane. The gravitational torque increases from the initial position until the horizontal axis is reached and then decreases towards the target. Furthermore, for all sessions, the inertia of the arm was systematically varied by applying additional weights to its extremity. This imposed further changes in the magnitudes of the gravitational torques. We tested to which extent the simulated and real robot arm were able to perform accurate pointing movements under these experimental conditions. Performance of the simulated or real robot arm was assessed after learning.

### Simulation and robotic experiments

Note that the learning phase was carried out only during the session I and for the 3 (i.e. 10°, 20° and 30°) of the 8 amplitudes. In addition, learning took place under different mass conditions. In the vertical plane the increase of the inertia of the moveable segment implies changes in both inertial and gravity torques and constitutes a good example for testing the capacity of our model to integrate both intrinsic (inertia) and extrinsic (gravity) parameters. Therefore, the capacity of generalization of our cerebellar neural network was tested in two ways: 1/In the first session, the performance in the other amplitudes than those used for training were assessed, i.e. interpolations to 15° and 25° and extrapolations to 5°, 35° and 40° for up and down directions. 2/In the second and third session, by quantifying (the difference between desired and performed) movements which differed significantly from those imposed during the learning procedure in the first session. Note that while adaptations to changes in gravitational force can be tested empirically, the initial learning of internal representations of gravity can (currently) only be studied by means of simulations and robotic experiments using anthropomorphic limbs.

### Simulated task

#### Simulation 1

Learning took place in the first session under three mass conditions: no additional mass (this condition was labeled M0; weight of the movable segment: 0.4 Kg), addition of a mass at the extremity of the segment corresponding to an increase of 12.5% of its inertia (labeled M1), and a mass that increased the inertia of the segment by 25% (labeled M2). A separate training session was performed for each mass, resulting in three weight matrices. Then the performance of pointing movements and the capacity for inter- and extrapolation was tested in session I, II and III. As a control, performance was also tested in a zero gravity environment.

#### Simulation 2

For this second simulation, we tested the capability of the model to generalize for different masses (i.e. to generalize for different gravitational torques) not used during the learning. A first one without any additional mass (labeled M0_T), and five others with additional masses corresponding respectively to an increase of 12.5% (M1_T, “T: Training”), 25% (M2_T), 30% (M3_T), 40% (M4_T), and 50% (M5_T) with respect to the M0_T condition. It is important to note that, in contrast to the simulation 1, motor learning was not performed for each mass separately, but by interchanging continuously the six masses throughout the training, which resulted in a single weight matrix. Then, the performance of pointing movements and the generalization capacity for inter- and extrapolation was tested in session I, II and III, under inertial conditions other than those used during training, i.e. with six different masses than those used during the training phase representing 5% (labeled M0_Iep, “Iep”: Intra-extrapolated positions), 18% (M1_Iep), 27.5% (M2_ Iep), 35% (M3_ Iep), 45% (M4_ Iep), and 60% (M5_ Iep) of the weight of the arm.

### Experimental procedure with the real robotic arm

The robot experiments were strictly identical to those performed during the “Simulation 1”. However, since the mass at the tip of the segment could not be changed after each trial for technical reasons, robot experiments similar to those presented during “Simulation 2” could not be performed.

### Data analysis

Motor performance was assessed by taking into account both the dynamic and static components of the movement. The static component was defined as the period when the displacement reached and remained within ±5% of its final value. The dynamic component was defined as the transient displacement from the movement onset up to the beginning of the static component. For the dynamic and static components, the performance was quantitatively assessed by the RMSE (Root Mean Square Error) between the desired and performed displacement according to the following formula:
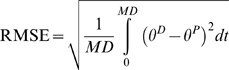
where MD is the movement duration, *θ^D^* and *θ^P^* represent the desired and performed angular displacement, respectively. The RMSE corresponding to the dynamic component of the displacement provided by the model was labeled RMSE_D_, the static component was labeled RMSE_S_. Whereas the RMSE_D_ can be considered as transient and related to the dynamics of the system (e.g. delays, stiffness), the RMSE_S_ is observed once the system reached its steady state and is similar to the constant errors analyzed in studies investigating arm pointing movements in humans (for a review see [23]).

In order to compare the effect of the experimental conditions (mass, amplitude, direction and initial position) on RMSE_D_ and RMSE_S_, a statistical analysis has been conducted. According to the normality of the distribution of the data (tested using a Lillefort's test) we used either parametric (e.g. *t*-test, ANOVA) or non-parametric (e.g. Wilcoxon, Kruskall-Wallis tests) statistical methods.

## Results

### Simulation 1

**Figure 4:**
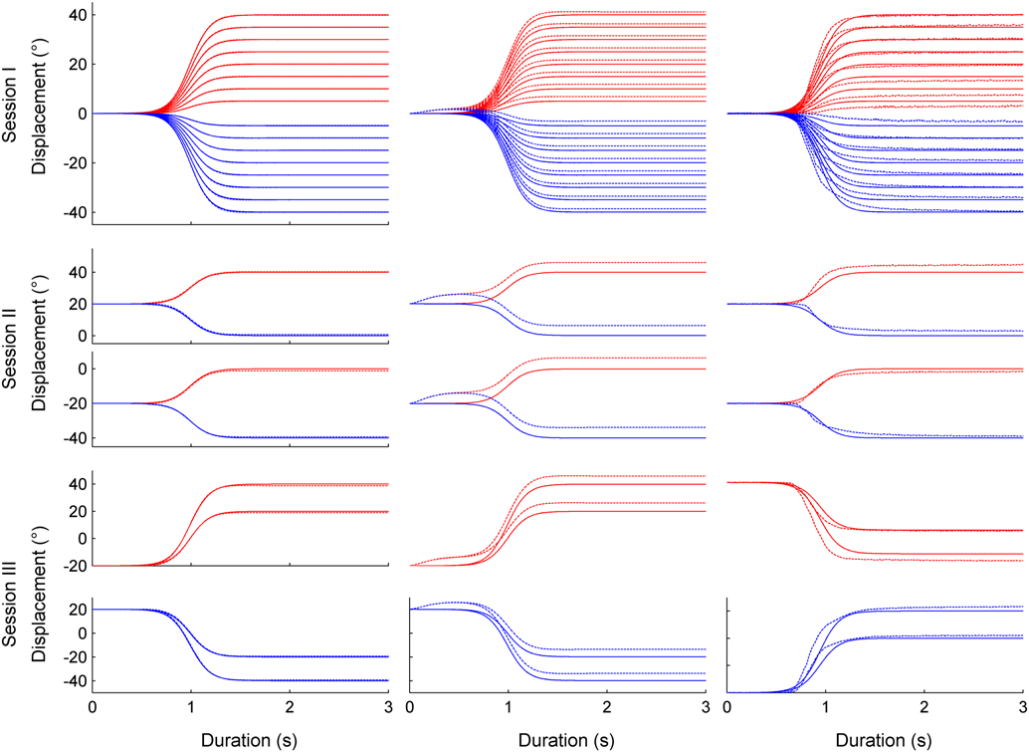
Performance of the model (M0 condition) for simulation 1 and the robotic experiment. Left column: arm movements performed during simulation 1 in the sagittal plane with no additional mass (M0) during the first (first row), second (second row) and third session (third row). Performed (dashed trace) and desired (continued trace) angular displacement. Red and blue traces: up and down movements respectively. Second column: Arm movements under zero gravity conditions after learning under normal gravity condition, Session I, II and III, no additional mass (M0). Third column: Robot arm movements in the sagittal plane with no additional mass (M0) during the first, second and third session.

The goal of the first simulation was to establish the performance of the proposed model after learning one specific weight matrix for each of the three mass conditions. Figure 4 (first column) shows performed (dashed trace) and desired (continuous trace) movement amplitudes over time for all movements and sessions under the mass condition M0. Figure 4A depicts the upward (red) and downward (blue) movements for the session I (first row) for the session II (second row) and for the session III (third row). Clearly, the network successfully learned to control movements against and with gravity.

#### Motor performance after learning (under training conditions)

Table 1 (column ‘Training’) shows that after learning the overall performance of our model was good; the average RMSE_D_ and RMSE_S_ between the desired and performed movement ranged between 0.04° and 0.08°. Statistical analysis did not reveal any significant effect (*t*-tests p>0.05). Notably, there was no significant difference between RMSE_D_ and RMSE_S_ errors. Interestingly, values of RMSE_D_ and RMSE_S_ were similar for the three weight conditions (matrixes). Furthermore, movement direction had no significant impact on the RMSE_D_ (up: 0.07±0.02°; down: 0.06±0.02°) and RMSE_S_ (up: 0.05±0.02°; down: 0.07±0.02°) values.

**Table 1:** Pointing errors for simulation 1.

		Training	Iep	Iep	Iep	Average Iep
		SI	SI	SII	SIII	
**M0**	**D**	0.08+0.02	0.09+0.02	0.02+0.01	0.10+0.05	0.08±0.04
**M0**	**S**	0.06+0.01	0.08+0.03	0.14+0.02	0.37+0.27	0.16±0.17
**M1**	**D**	0.05±0.01	0.06±0.02	0.02±0.01	0.08±0.05	0.06±0.03
**M1**	**S**	0.06±0.01	0.07±0.03	0.14±0.02	0.37±0.18	0.15±0.15
**M2**	**D**	0.04±0.02	0.05±0.03	0.02±0.01	0.08±0.03	0.05±0.03
**M2**	**S**	0.06±0.02	0.07±0.04	0.14±0.01	0.37±0.11	0.15±0.13
**Average M0–2**	**D**	0.06±0.02	0.07±0.03	0.02±0.01	0.09±0.04	
**Average M0–2**	**S**	0.06±0.02	0.07±0.03	0.14±0.02	0.37±0.20	

Average RMSE_D_ (D) and RMSE_S_ (S) for each mass condition for the session I (SI), II (SII) and III (SIII). Training: Training set. Iep (inter- and extrapolated positions): test set. Average Iep: RMSE values for the test set averaged across SI, SII and SIII. Average M0–2: RMSE_D_ and RMSE_S_ values for both the training and test set averaged across the different mass conditions (M0, M1, M2) for each session (SI, SII, SIII).

#### Motor performance for novel movements (intra- and extrapolation)

Table 1 (column ‘Average Iep’) shows that novel movements were less precise than those included in the sample used for training. This poorer motor performance was reflected by the RMSE_S_ (*t*-test, p<0.001) which increased by a factor of three (to about 0.15°) over the three sessions, while the RMSE_D_ values remained at those obtained for the training set (about 0.06°, (*t*-test, p>0.45)). However, the difference between RMSE_D_ and RMSE_S_ was not statistically significant (Wilcoxon test, p>0.26), likely due to the large variability between pointing conditions. As in the training condition, there was no difference between movements with the three masses (i.e. between the three matrices of weights) (Wilcoxon test, p>0.13). However, the RMSE_S_ clearly varied between the three sessions: the performance of intra- and extrapolation within session I was similar to the performance in the training set (on average 0.07±0.03°), but increased to 0.14±0.02° in session II and increased even more in session III (0.37±0.20°). Note that this was not the case for RMSE_D_ (0.07±0.03°; 0.02±0.01°; 0.09±0.04°).

#### Motor performance under 0 g

The integration of gravitational effects by the network after the learning phase was assessed by examining RMSE_D_ and RMSE_S_ values when exposing the model to a 0-gravity environment. Figure 4 (second column) depicts movement traces in 0 g after the model had been trained to control the arm in 1 g. In 0 g the RMSE_D_ and RMSE_S_ values were about 38 and 22 times higher than their counterparts obtained under normal gravity conditions (Wilcoxon test, p<0.001 all conditions considered). They ranged, depending on the sessions and weight conditions, between 1.54° and 5.64° for RMSE_S_ and between 1.17° and 6.30° for RMSE_D_.

### Simulation 2

The goal of Simulation 2 was to examine motor performance after having learned a single weight matrix, which had, in addition, to cover a wider range of mass conditions (6 different masses up to a 50% increase). The generalization over these different mass conditions was tested.

#### Motor performance after learning (under training conditions)

The grand average RMSE_S_ (for various masses and movement amplitudes) was 0.48±0.32°, that is, larger than for simulation 1 (*t*-test, p<0.001). Similarly, the grand average of the RMSE_D_ increased to 0.44±0.30° compared to simulation 1 (*t*-test, p<0.001) but was not significantly different from the RMSE_S_ (Wilcoxon test, p>0.60). However, and in contrast to simulation 1, the performance depended of the mass. Table 2 (column ‘Training’) shows the average RMSE_S_ and RMSE_D_ for each mass condition. Clearly, the best performance (i.e. lowest RMSEs and RMSE_D_) was found for intermediate masses (M2_T, M3_T), while the performance for small (M0_T, M1_T) or great masses (M4_T, M5_T) significantly increased. These differences were statistically significant (Kruskall-Wallis test, p<0.001).

**Table 2:** Pointing errors for simulation 2.

		Training			Iep	Iep	Iep	Average Iep
		SI			SI	SII	SIII	
**M0_T**	**D**	0.93+0.01	**M0_Iep**	**D**	0.93±0.01	0.05±0.01	0.13±0.06	0.55±0.42
**M0_T**	**S**	1.01+0.05	**M0_Iep**	**S**	0.98±0.08	0.16±0.03	0.40±0.31	0.67±0.39
**M1_T**	**D**	0.53+0.01	**M1_Iep**	**D**	0.53±0.01	0.05±0.01	0.12±0.05	0.33±0.22
**M1_T**	**S**	0.59+0.04	**M1_Iep**	**S**	0.58±0.06	0.15±0.01	0.39±0.15	0.44±0.19
**M2_T**	**D**	0.15+0.02	**M2_Iep**	**D**	0.14±0.02	0.05±0.01	0.14±0.03	0.12±0.04
**M2_T**	**S**	0.17+0.04	**M2_Iep**	**S**	0.18±0.05	0.15±0.02	0.38±0.09	0.22±0.11
**M3_T**	**D**	0.05+0.02	**M3_Iep**	**D**	0.06±0.03	0.06±0.01	0.15±0.03	0.08±0.05
**M3_T**	**S**	0.05+0.03	**M3_Iep**	**S**	0.05±0.04	0.15±0.03	0.38±0.13	0.15±0.15
**M4_T**	**D**	0.35+0.03	**M4_Iep**	**D**	0.35±0.04	0.07±0.01	0.17±0.03	0.25±0.12
**M4_T**	**S**	0.33+0.05	**M4_Iep**	**S**	0.31±0.08	0.15±0.05	0.38±0.25	0.29±0.16
**M5_T**	**D**	0.66+0.04	**M5_Iep**	**D**	0.66±0.05	0.08±0.01	0.20±0.04	0.43±0.27
**M5_T**	**S**	0.67+0.07	**M5_Iep**	**S**	0.62±0.11	0.16±0.07	0.41±0.35	0.47±0.27
**Aver M0–5_T**	**D**	0.44+0.30	**Aver M0–5_Iep**	**D**	0.44±0.30	0.06±0.01	0.15±0.05	
**Aver M0–5_T**	**S**	0.48+0.32	**Aver M0–5_Iep**	**S**	0.45±0.32	0.15±0.04	0.39±0.24	

Average RMSE_D_ (D) and RMSE_S_ (S) for each mass condition for the session I (SI), II (SII) and III (SIII). Training: Training set. Iep (inter- and extrapolated positions): test set. M_i__T (0≤i≤5): masses used during the training set. M_i__Iep (0≤i≤5): masses used during the test set. Average Iep: RMSE values for the test set averaged across SI, SII and SIII. Aver M0–5_T, Aver M0–5_T; Aver M0–5_Iep: RMSE_D_ and RMSE_S_ values for the training (_T) and test set (_Iep) averaged across the different mass conditions, respectively.

#### Motor performance for novel movement (intra- and extrapolation)

**Figure 5:**
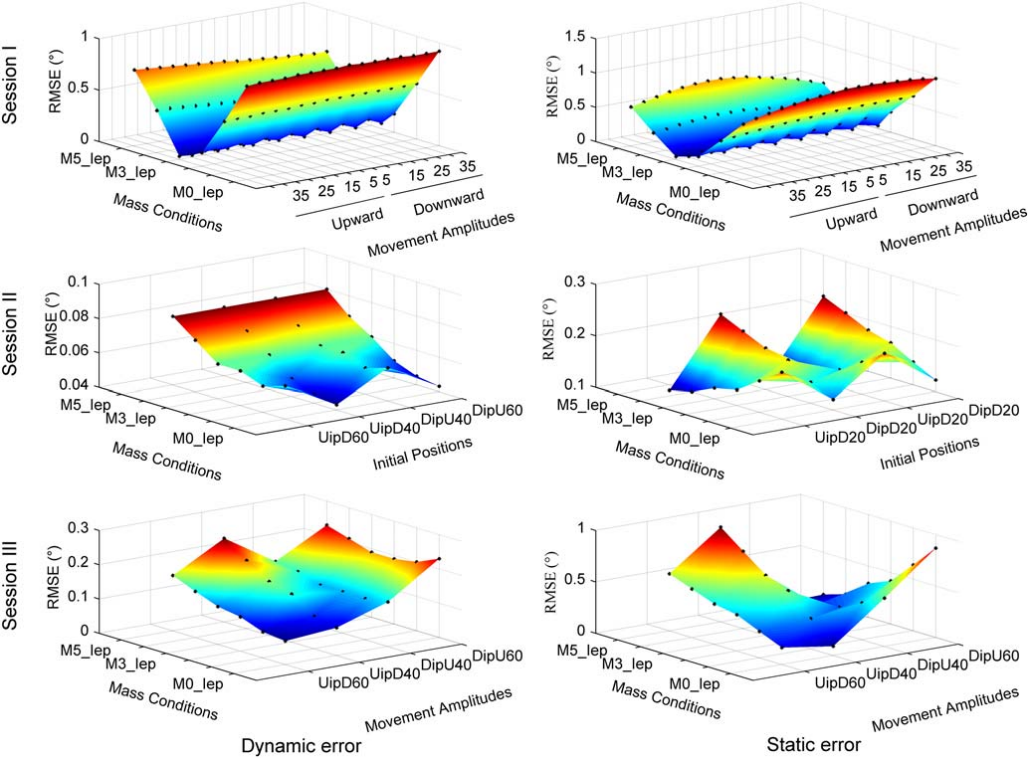
Performance of the model for simulation 2. Pointing error as a function of weight (mass condition M0_Iep, M1_Iep, M2_Iep, M3_Iep, M4_Iep and M5_Iep) and movement amplitude (and initial position). First column: dynamic error (RMSE_D_). Second column: static error (RMSE_S_). Each black dot represents an error measure (dynamic or static) obtained for a given experimental condition. IP: initial position.

Table 2 shows the average RMSE_D_ and RMSE_S_ for each mass condition and experimental session. Similar to the training session, the average performance was better for intermediate weights and poorer for light and heavy weights. Figure 5 shows the RMSE_D_ (left column) and RMSE_S_ (right column) for each of the three sessions. Each graph depicts the RMSE as a function of mass condition (i.e. for intra- and extrapolated masses) and as a function of movement amplitude and initial position.

Session I: with values of 0.44±0.30° and 0.45±0.32°, the grand averages (i.e. including all experimental conditions) of RMSE_D_ and RMSE_S_ were similar to the training condition (Table 2). As in the training condition, both RMSE_D_ and RMSE_S_ varied as a function of mass. This is illustrated in Figure 5 top row, which shows a V-profile of the RMSE, indicating that the RMSE varied as a function of mass, but not as a function of movement amplitude or movement direction. This was statistically confirmed using a Kruskall-Wallis test coupled with a multiple comparisons test for each factor (for both type of error; mass: p<0.001, amplitude and direction: p>0. 95).

Session II: the grand averages of RMSE_D_ (0.06±0.01°) and RMSE_S_ (0.15±0.04°) were smaller than those in session I (*t*-test, p<0.001) and smaller than those in the training session (Table 2). Figure 5 (middle row, note reduced Z-axis scale) shows that the RMSE_D_ varied little (p>0.10 for all comparisons) whereas the RMSE_S_ clearly varied as a function of mass and of movement direction (p<0.0014) but not of initial position and direction (p>0.70).

Session III: the grand averages of RMSE_D_ (0.15±0.05°) and RMSE_S_ (0.39±0.24°, Table 2) tended to be smaller than those in session I (*t*-test, p<0.01, only for the RMSE_D_) but larger than those in session II (*t*-test, p<0.01, for both type of RMSE). Figure 5 (bottom row) shows changes of RMSE_D_ and the RMSE_S_ as a function of mass, of movement amplitude and initial position. In both cases, the largest RMSE was generally found for movements of large amplitudes and either heavy or light masses. However, no significant effect of the mass, movement amplitude and initial position on both type of error was found (p>0.23 for all comparisons, except for the case where the RMSE_D_ was higher for a U movement of 60° amplitude than for a D movement of 40° amplitude (p = 0.009).

### Robot experiments

The aim of the robot experiments was to verify whether the simplified calculations of the gravity torque acting on the segment would be adequate for controlling an anthropomorphic robot arm. Only the equivalent of simulation 1 was performed with the robot. Figure 4 (right column) shows the movement of the robot arm for all movements and sessions under mass condition M0.

#### Motor performance after learning (under training conditions)

Table 3 (column ‘Training’) shows that the overall robot performance was not as accurate as for the simulated movements. Over all three mass conditions the average RMSE_D_ (1.51±0.71°) was higher (*t*-test, p<0.05) than the RMSE_S_ (1.08±1.01°). Table 3 shows that the RMSE_D_ and RMSE_S_ did not vary as a function of mass, of amplitude or movement direction (Kruskall-Wallis test coupled with a multiple comparison, p>0.14 for all comparisons).

**Table 3:** Pointing errors for robot experiments (conditions equivalent to simulation 1).

		Training	Iep	Iep	Iep	Average Iep
		SI	SI	SII	SIII	
**M0**	**D**	1.22±0.47	1.75+0.55	1.86±0.60	4.34±1.03	3.35±1.27
**M0**	**S**	0.83±0.79	0.95+0.59	2.60±1.34	2.87±1.78	1.75±1.45
**M1**	**D**	1.36±0.78	1.91±0.89	1.26±0.44	3.91±1.45	2.21±1.36
**M1**	**S**	1.04±1.21	1.34±1.11	1.25±0.43	2.55±1.92	1.59±1.35
**M2**	**D**	1.95±0.61	2.37±1.09	2.25±1.15	3.70±1.43	2.64±1.32
**M2**	**S**	1.37±0.89	1.47±0.95	3.61±2.27	3.93±1.84	2.49±1.93
**Average M0–2**	**D**	1.51±0.71	2.01±0.91	1.79±0.89	3.99±1.34	
**Average M0–2**	**S**	1.08±1.01	1.25±0.93	2.49±1.82	3.12±1.94	

Average RMSE_D_ (D) and RMSE_S_ (S) for each mass condition for the session I (SI), II (SII) and III (SIII). Training: Training set. Iep (inter- and extrapolated positions): test set. Average Iep: RMSE values for the test set averaged across SI, SII and SIII. Average M0–2: RMSE_D_ and RMSE_S_ values for both the training and test set averaged across the different mass conditions (M0, M1, M2) for each session (SI, SII, SIII).

#### Motor performance for novel movements (intra- and extrapolation)

Table 3 (column ‘Average Iep’) shows that performance for novel movements were less precise than those included in the sample used for training. However, the decrease was not statistically significant (*t*-test, p>0.10). As in the training condition, there was no significant difference between the three mass conditions (i.e. between the three matrixes). Furthermore, contrary to the simulation results, the RMSE_D_ and RMSE_S_ did not vary as function of the session. RMSE_D_ (averaged over the three weight conditions) for session I, II and III were respectively 2.01°±0.91, 1.79°±0.89 and 3.99°±1.34° while for RMSE_S_, they were respectively 1.25°±0.93°, 2.49°±1.82 and 3.12°±1.94°. Lastly, motor performance of the robot arm did not vary as a function of movement direction.

**Figure 6:**
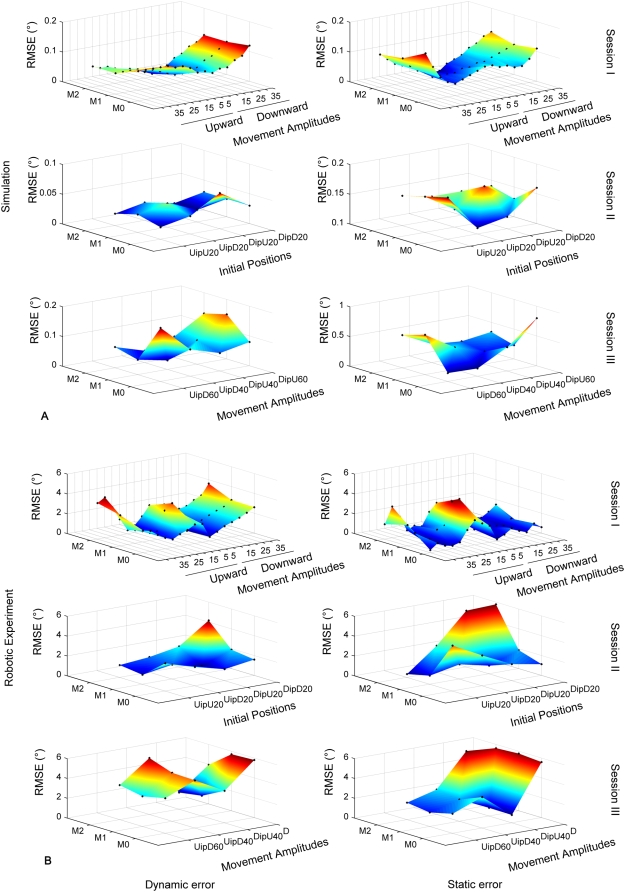
Comparison between simulated and robot movements. Distribution of the RMSE_D_ (left column) and RMSE_S_ (right column) for the three sessions for the simulation 1 (A) and the robotic experiment (B). Both type of error are represented as a function of movement amplitudes during the session I (i.e. intra- and extrapolated positions) and of initial positions and movement amplitudes during respectively the session II and III.

Figure 6 compares the performance of simulated and robot movements. Figure 6A shows RMSE_D_ and RMSE_S_ as a function of mass and movement amplitude conditions in session I (i.e. Inter and extrapolated position). For session II and III, RMSE_D_ and RMSE_S_ are illustrated as a function of initial position, movement amplitude and mass condition. Figure 6B shows the data for the corresponding robot movements. As already shown in Figure 4, the robot experiment produced errors larger than those in the corresponding simulation 1. Moreover, Figure 6 shows that errors on the three movement variables (amplitude, initial positions and directions of the movements) tended to be qualitatively different between the robot experiment and the simulation. For the simulation in session 1, maximal dynamic and static errors were found for the largest movement amplitudes (e.g. 40° up and down), independently of the mass condition. Although the robot experiment replicated the independence on the mass condition, the dependence on amplitude changed: largest and smallest movement amplitudes provoked maximal errors (e.g. 40° as well as 5° and 10°). For session II, except for the absolute values, no striking qualitative difference was found between simulation and robot experiment. In session III, the simulation showed maximal errors preferentially for large movement amplitudes and specific mass conditions. In contrast, the robot experiment seemed to be less sensitive to the mass condition and produced less symmetrical error surfaces.

## Discussion

The aim of this study was to investigate whether a command circuit, deduced from mathematical calculation and schematically comparable to the cerebellar pathways would learn the inverse dynamics of an anthropomorphic robot arm during vertical pointing movements. For this purpose, we varied arm inertia (by means of additional masses), arm initial position, movement amplitude and direction and obtained gravitational and inertial torques which changed in a highly non-linear and even non-monotonic fashion. The results from both simulations and robot experiments show that this model through learning acquired the dynamics of the limb, namely an inverse model of the inertial and gravitational torques.

After learning, sensory and motor signals were processed in a predictive neural network (embedding the internal model of the dynamics of the limb), so that adequate neural commands were computed to perform arm pointing movements in the sagittal plane. Interestingly, this skill was generalized to movements different from those previously learned, although movements that required interpolations and extrapolations from the training set were in general performed less precisely. Not surprisingly, the pointing errors were higher for the actual than for the simulated robotic arm. Nevertheless, the robot experiments showed that the approximations of the model, in particular the calculation of gravity torques were adequate and sufficient to control the one DoF robot arm.

### The cerebellum computes both sensorimotor predictions and dynamic inverse computations

In our model, sensory and motor signals are used to predict the gravitational torque exerted on the arm and then to compute inverse dynamics of the arm. This is in agreement with previous studies which have suggested that the cerebellum implements an inverse model to perform the dynamic and/or kinematic inverse computation [9], [10], [24], [25]. Moreover, the proposed model also reinforces the idea proposed by prior investigations that the cerebellum computes sensorimotor predictions [11], [12], [26]–[28]. For instance, it has been suggested [29] that the cerebellum incorporates a forward model that predicts the sensory consequences of movements. The authors concluded that the cerebellum was involved in sensorimotor prediction and was therefore a plausible neural site to implement a forward model. Our model also supports the idea that the cerebellum includes both forward and inverse models [30]–[32], and it provides an anatomical plausibility and sensorimotor learning scheme for both internal models. Indeed, by taking into account the functional and the anatomical features of the cerebellar pathways, our model provides a coherent theoretical framework that implements the coexistence of these two internal models replicating both the functional role of the cerebellar cortex and its corresponding anatomical structure. Specifically, it shows that the cerebellar cortex could possibly implement a predictive neural network, which is equivalent to a forward model in order to predict the effects of the gravitational and inertial torques applied to the segment. In our model, this prediction is propagated through the cerebellar connectivity via three biologically plausible internal feedback loops: a first one in the cerebellar cortex and the two others in the cerebellar pathways. This model suggests that the cerebellar cortex is primarily responsible of sensorimotor predictions, whereas the entire cerebellum is in charge of the inverse dynamics computation. Hence, it is compatible with previous studies that have proposed that the cerebellum integrates an inverse model, as well as with studies that have suggested a predominant role of the cerebellum in sensorimotor prediction.

Computing an inverse model from a forward model is a technique used for industrial plant control [33] and has already been applied to motor control [12], [34], however, the proposed circuits were hardly biological plausible. On the contrary, the significance of the present model is that its structure is deduced from physical constraints, and it is consistent with the cerebellar connectivity. It controls arm pointing movements in a vertical plane based on the acquisition of a direct predictive model of the limb biomechanics, which is sufficient to approximate the inverse dynamics via multiple looped structures. It must also be noted that the principle of using such multiple recurrent loops, particularly through the forward model, makes our cerebellar model somewhat comparable to recurrent neural networks, reinforcing thus, the idea that this type of neural network structure is able to correctly simulate dynamic behavior [35]–[38]. Indeed, although these studies developed neural models without specifically addressing any particular anatomical structures; they revealed that recurrent neural networks efficiently learned temporal patterns by feeding back the copies of the current sensorimotor prediction outputs to the next sensorimotor inputs. Finally, one important theoretical and computational consequence of the architecture of this model is that it offers a biologically plausible solution that avoids the well-known, but artificial two-step sensorimotor learning scheme: most published models require, first, the learning of the forward model [34], or the assumption that the forward model is *a priori* known, and subsequently the learning of the inverse model [39], [40].

### The importance of sensorimotor prediction of gravitational torques in neural control

Our model approximates inverse dynamics by means of predictions, i.e. by a forward model based on sensory and pre-motor signals. This internal forward model is acquired through motor learning and provides a control for vertical arm movements.

Previous studies, using simulation [34] or experimentation [7], [8], have highlighted the importance of internal forward models for sensorimotor predictions. For instance, using an object manipulation task, it has been shown that subjects learned sensorimotor predictions prior to the control of objects; the learning of a forward model precedes that of an inverse model [41]. Similarly, by means of simulations, it has been shown that even an approximate internal forward model was able to train an inverse model [34]. Furthermore, other investigations [8], [42], using a motor imagery paradigm, asked subjects to execute or to imagine horizontal and vertical single-joint arm pointing movements, and showed that movement durations were very similar for both conditions. Isochrony between executed and imagined movements is achieved by well trained internal forward models which precisely predict the gravito-inertial forces acting on the arm. More recently it has been shown that subjects improved 3D arm pointing movements during a speed/accuracy trade-off during mental practice [43]. The authors suggested that an efferent copy of the neural drive during motor imagination would be available to the forward model which, in turn, was thought to train the inverse model. These studies are in accordance with the present results indicating a major role of the forward model in motor control and learning.

Moreover, models of physical laws that are able to predict object motion in the gravitational field may also be represented in the brain [3] (for a review see [2]). For instance, during ball catching in the gravitational field, it has been found that the vestibular network and the medial cerebellum were activated [44] (for the implication of the vestibular system see [45]–[47]). It must be noted that the predictive mechanisms embedded in our cerebellar model is not in contradiction with the idea that the posterior parietal cortex could also be involved in motor predictions. Since both parietal cortex and cerebellum are anatomically connected through a functional loop [48], [49], they could share a neural substrate for forward models and may have different roles in motor prediction [50], [51]. Thus, prediction related to a high cognitive level could be implemented in the parietal cortex, whereas sensorimotor predictions (e.g. self-generated movements) may be performed by the cerebellum [12], [50].

Beyond the cerebellum and the parietal posterior cortex that seem to be the main candidates for forward models [31], [50], others studies, without always mentioning explicitly such a computational concept, revealed that others neural regions would also incorporate predictive mechanisms. For instance, the basal ganglia that projects to the cerebral cortex through multiple parallel channels [52], [53] would also perform predictions based on the environmental states to select the appropriate action in a given context [54], [55]. Also, highly neural recurrent structures such as the hippocampus [56], [57], the prefrontal [58], [59] and the orbitofrontal [60], [61] cortex seem to perform associative prediction that would reflect outcome expectancies, providing thus, an internal representation of the consequences likely to follow a particular act.

However, two main differences appeared between these predictive mechanisms and those embedded in the cerebellum and by extension in our model. First, our cerebellar model used a learning rule that simulated supervised learning that takes place in the cerebellum and driven by the climbing fibers messages issued from the inferior olive. Here, pointing errors were detected and used to compute the teaching signals, as it seems to occur in the inferior olive. This type of learning contrasts with the reinforcement learning used in the basal ganglia [54], [55] or hippocampus [57] and the unsupervised learning that takes place in the cortex [54]. However, more generally and independently of the learning procedure used, a key feature of our model is its capability to compute an inverse mapping by means of a direct mapping embedded into recurrent loops. Therefore, we can wonder if it might be possible that similar mechanisms, albeit a different learning method, might take place in others predictive structures as those mentioned to compute inverse mappings. Second, our cerebellar model predicts the sensorimotor output of the system contrary to the predictive mechanisms above described that perform predictions at a higher and more general behavioral level. Such lower and higher levels of prediction have been investigated in a series of computational studies in order to understand their interactions using multiples environments and tool manipulations [30], [32], [38], [62], [63]. The lower level would correspond to the sensorimotor processes of detailed environment interactions to provide an accurate control of limb motion. The higher cognitive level would embed abstractions of those lower sensorimotor processes level to infer behaviors or plan goal-oriented movements.

Our cerebellar model implemented predictive mechanisms related to the sensorimotor (short-timescale) predictions of the internal state of the arm, addressing thus, mainly the lower level. However, it is of interest to note that our model predicts the internal state of the arm (inertia) but also its mechanical interaction with the external (gravity) context in which actions takes place. In humans, the estimation of action context in terms of mechanical interaction between the body limb and the environment or a tool is essential for the performance of skilful movements. For instance, when we make a reaching movement while rotating our torso, we compensate for the velocity-dependent Coriolis forces that arise from the rotation and act on our arm [64]. Likewise, the development of a new forward model in the context of microgravity, allows astronauts to adapt their actions during space flights. Therefore, from a general point of view, the cerebellar model presented here takes in to accounts these dynamical interactions between a given environment and the body limbs but is not able to deal with multiple environments or tools. Thus, this model could be naturally embedded into a more general neural structure including a higher level. For instance, this could be explicitly done by means of a modular structure incorporating switching mechanisms such as gate-selection [30], [32], [38], or by means of an emerging functional hierarchy using neural networks with neurons having different timescale [63].

### Performance and limits of our model of the cerebellar pathways

The movement accuracy achieved by our model during the robot experiment (RMSE_S_ between 0.33° and 5.81°) was comparable to previously reported data for human arm pointing movements with one [5] or two [65] geometrical DoF, with constant pointing errors less than 5°. The model was able to learn a set of upward and downward movements and to generalize by interpolation and extrapolation to other types of movements including those with non-monotonous profiles of gravitational torque. However, the pointing errors varied as a function of the type of generalization. First, the error increased when movement amplitudes were tested outside those of the training range. Second, the error also varied as a function of the time-varying profile of gravitational torque exerted during the movement: monotonic variations of gravitational torque (session I and II) provoked smaller errors than non-monotonic variations (session III). Furthermore, although learning was faster and produced smaller errors when based on a specific weight matrix per inertial condition, a global weight matrix across different inertial conditions provided much better generalization.

As previously mentioned, the two types of errors presented here refer to two different aspects of the performance of the model. Therefore, a reduced dynamic error reflects the capacity of our model to take into account the dynamic features of the system (e.g. non-linearities, delays, stiffness) whereas a small static or constant error would suggest the presence of a small and constant bias in the sensorimotor transformations [23]. Concerning the simulations 1 and 2, our model of cerebellar pathways was able to capture adequately the dynamics of the moving limb. However, the further the model had to extrapolate from the training conditions, the stronger was the constant bias in the sensorimotor transformations (even if both types of error remain small, <1.2°). Furthermore, whereas for the two simulations the dynamic error was always inferior or equivalent to the static error, during the robotic experiment session this trend was inverted (dynamic higher than static error). This is due to the fact that contrary to the simulation, when the learning is performed during the experimental session, some mechanical features (e.g. stiffness) were either not accurately taken into account or neglected by the model of cerebellar pathways.

### Conclusions

This study presents a command circuit comparable to the cerebellar pathways that learns the inverse dynamics of an anthropomorphic robot arm, including the effects of the gravitational forces. Learning was achieved through an internal forward model allowing the computation of an approximation of the inverse dynamics. After learning, this circuit was able to drive arm movements in the vertical plane, with an accuracy comparable to that of human movements. The model suggests that the cerebellar cortex is a plausible neural site for learning internal predictive forward models of the gravitational forces, and that the whole cerebellum is likely able to perform approximate inverse computations.

## References

[1] Pozzo T, Papaxanthis C, Stapley P, Berthoz A (1998). The sensorimotor and cognitive integration of gravity.. Brain Res Rev.

[2] Zago M, Lacquaniti F (2005). Visual perception and interception of falling objects: a review of evidence for an internal model of gravity.. J Neural Eng.

[3] McIntyre J, Zago M, Berthoz A, Lacquaniti F (2001). Does the brain model Newton's laws?. Nat Neurosci.

[4] Papaxanthis C, Pozzo T, Stapley P (1998). Effects of movement direction upon kinematic characteristics of vertical arm pointing movements in man.. Neurosci Lett.

[5] Gentili R, Cahouet V, Papaxanthis C (2007). Motor planning of arm movements is direction-dependent in the gravity field.. Neuroscience.

[6] Cheron G, Bengoetxea A, Pozzo T, Bourgeois M, Draye JP (1997). Evidence of a preprogrammed deactivation of the hamstring muscles for triggering rapid changes of posture in humans.. Electroencephalogr Clin Neurophysiol.

[7] Flanagan JR, Lolley S (2001). The inertial anisotropy of the arm is accurately predicted during movement planning.. J Neurosci.

[8] Gentili R, Cahouet V, Ballay Y, Papaxanthis C (2004). Inertial properties of the arm are accurately predicted during motor imagery.. Behav Brain Res.

[9] Kawato M, Furukawa K, Suzuki R (1987). A hierarchical neuronal network model for control and learning of voluntary movement, inputs into the cerebellar cortex.. Biol Cybern.

[10] Schweighofer N, Arbib MA, Kawato M (1998). Role of the cerebellum in reaching movements in humans. I. Distributed inverse dynamics control.. Eur J Neurosci.

[11] Miall RC, Weir DJ, Wolpert DM, Stein JF (1993). Is the cerebellum a Smith predictor?. J Mot Behav.

[12] Wolpert DM, Miall RC (1996). Forward Models for Physiological Motor Control.. Neural Netw.

[13] Maschke M, Gomez CM, Ebner TJ, Konczak J (2004). Hereditary cerebellar ataxia progressively impairs force adaptation during goal-directed arm movements.. J Neurophysiol.

[14] Diedrichsen J, Hashambhoy Y, Rane T, Shadmehr R (2005). Neural correlates of reach errors.. J Neurosci.

[15] Eskiizmirliler S, Forestier N, Tondu B, Darlot C (2002). A model of the cerebellar pathways applied to the control of a single-joint robot arm actuated by McKibben artificial muscles.. Biol Cybern.

[16] Ebadzadeh M, Tondu B, Darlot C (2005). Computation of inverse functions in a model of cerebellar and reflex pathways allows to control a mobile mechanical segment.. Neuroscience.

[17] Jordan MI, Wolpert DM, Gazzaniga MS (1999). Computational motor control.. The Cognitive Neurosciences.

[18] Ebadzadeh M, Darlot C (2003). Cerebellar learning of bio-mechanical functions of extra-ocular muscles: modeling by artificial neural networks.. Neuroscience.

[19] Darlot C, Zupan L, Etard O, Denise P, Maruani A (1996). Computation of inverse dynamics for the control of movements.. Biol Cybern.

[20] Eccles JC, Ito M, Szentagothai J (1967). The cerebellum as a neuronal machine.

[21] Ito M (2006). Cerebellar circuitry as a neuronal machine.. Prog Neurobiol.

[22] Tondu B, Lopez P (2000). Modelling and control of McKibben artificial muscle robot actuators.. IEEE Control Systems Magazine.

[23] McIntyre J, Stratta F, Droulez J, Lacquaniti F (2000). Analysis of pointing errors reveals properties of data representations and coordinate transformations within the central nervous system.. Neural Comput.

[24] Kawato M, Gomi H (1992). The cerebellum and VOR/OKR learning models.. Trends Neurosci.

[25] Spoelstra J, Schweighofer N, Arbib MA (2000). Cerebellar learning of accurate predictive control for fast-reaching movements.. Biol Cybern.

[26] Blakemore SJ, Frith CD, Wolpert DM (2001). The cerebellum is involved in predicting the sensory consequences of action.. NeuroReport.

[27] Kawato M, Kuroda T, Imamizu H, Nakano E, Miyauchi S (2003). Internal forward models in the cerebellum: fMRI study on grip force and load force coupling.. Prog Brain Res.

[28] Rost K, Nowak DA, Timmann D, Hermsdorfer J (2005). Preserved and impaired aspects of predictive grip force control in cerebellar patients.. Clin Neurophysiol.

[29] Blakemore SJ, Wolpert DM, Frith CD (1998). Central cancellation of self produced tickle sensation.. Nat Neurosci.

[30] Wolpert DM, Kawato M (1998). Multiple paired forward and inverse models for motor control.. Neural Netw.

[31] Wolpert DM, Miall RC, Kawato M (1998). Internal models in the cerebellum.. Trends in Cognitive Sciences.

[32] Haruno M, Wolpert DM, Kawato M (2001). Mosaic model for sensorimotor learning and control.. Neural Comput.

[33] Datta A (1998). Adaptive Internal Model Control.

[34] Jordan MI, Rumelhart DE (1992). Supervised learning with a distal teacher.. Cognitive Science.

[35] Elman J (1990). Finding structure in time.. Cognitive Science.

[36] Jordan M (1986). Attractor dynamics and parallelism in a connectionist sequential machine..

[37] Tani J, Ito M (2003). Self-Organization of Behavioral Primitives as Multiple Attractor Dynamics: A Robot Experiment.. IEEE Transaction on Systems, Man, and Cybernetics - Part A: Systems and Humans.

[38] Tani J (2007). On the interactions between top-down anticipation and bottom-up regression.. Front Neurorobotics.

[39] Bullock D, Grossberg S, Guenther F (1993). A Self-Organizing Neural Model of Motor Equivalent Reaching and Tool Use by a Multijoint Arm.. Journal of Cognitive Neuroscience.

[40] Grosse-Wentrup M, Contreras-Vidal JL (2007). The role of the striatum in adaptation learning: a computational model.. Biol Cybern.

[41] Flanagan JR, Vetter P, Johansson RS, Wolpert DM (2003). Prediction precedes control in motor learning.. Curr Biol.

[42] Papaxanthis C, Schieppati M, Gentili R, Pozzo T (2002). Imagined and actual arm movements have similar durations when performed under different conditions of direction and mass.. Exp Brain Res.

[43] Gentili R, Papaxanthis C, Pozzo T (2006). Improvement and generalization of arm motor performance through motor imagery practice.. Neuroscience.

[44] Indovina I, Maffei V, Bosco G, Zago M, Macaluso E (2005). Representation of visual gravitational motion in the human vestibular cortex.. Science.

[45] Ito M (1984). The cerebellum and neural control.

[46] Manzoni D (2005). The cerebellum may implement the appropriate coupling of sensory inputs and motor responses: evidence from vestibular physiology.. Cerebellum.

[47] Manzoni D (2007). The cerebellum and sensorimotor coupling: looking at the problem from the perspective of vestibular reflexes.. Cerebellum.

[48] Clower DM, West RA, Lynch JC, Strick PL (2001). The inferior parietal lobule is the target of output from the superior colliculus, hippocampus, and cerebellum.. J Neurosci.

[49] Glickstein M (2000). How are visual areas of the brain connected to motor areas for the sensory guidance of movement?. Trends Neurosci.

[50] Blakemore SJ, Sirigu A (2003). Action prediction in the cerebellum and in the parietal lobe.. Exp Brain Res.

[51] Imamizu H, Kuroda T, Yoshioka T, Kawato M (2004). Functional magnetic resonance imaging examination of two modular architectures for switching multiple internal models.. J Neurosci.

[52] Middleton FA, Strick PL (1998). Cerebellar output: motor and cognitive channels.. Trends Cognitive Science.

[53] Middleton FA, Strick PL (2000). Basal ganglia and cerebellar loops: motor and cognitive circuits.. Brain Res Rev.

[54] Doya K (2000). Complementary roles of basal ganglia and cerebellum in learning and motor control.. Curr Opin Neurobiol.

[55] Kawato M, Samejima K (2007). Efficient reinforcement learning: computational theories, neuroscience and robotics.. Curr Opin Neurobiol.

[56] Hölscher C, Jacob W, Mallot HA (2003). Reward modulates neuronal activity in the hippocampus of the rat.. Behav Brain Res.

[57] Okatan M (2009). Correlates of reward-predictive value in learning-related hippocampal neural activity.. Hippocampus.

[58] Barraclough DJ, Conroy ML, Lee D (2004). Prefrontal cortex and decision making in a mixed-strategy game.. Nat Neurosci.

[59] Tanaka SC, Doya K, Okada G, Ueda K, Okamoto Y, Yamawaki S (2004). Prediction of immediate and future rewards differentially recruits cortico-basal ganglia loops.. Nat Neurosci.

[60] Roesch MR, Olson CR (2004). Neuronal activity related to reward value and motivation in primate frontal cortex.. Science.

[61] Schoenbaum G, Chiba AA, Gallagher M (1998). Orbitofrontal cortex and basolateral amygdala encode expected outcomes during learning.. Nat Neurosci.

[62] Tani J, Nolfi S (1999). Learning to perceive the world as articulated: an approach for hierarchical learning in sensory-motor systems.. Neural Netw.

[63] Yamashita Y, Tani J (2008). Emergence of functional hierarchy in a multiple timescale neural network model: a humanoid robot experiment.. PLoS Comput Biol.

[64] Pigeon P, Bortolami SB, DiZio P, Lackner JR (2003). Coordinated turn-and-reach movements. I. Anticipatory compensation for self-generated coriolis and interaction torques.. J Neurophysiol.

[65] Soechting JF, Flanders M (1989). Errors in pointing are due to approximations in sensorimotor transformations.. J Neurophysiol.

